# Outbreak of *Fusarium oxysporum* infections in children with cancer: an experience with 7 episodes of catheter-related fungemia

**DOI:** 10.1186/s13756-017-0247-3

**Published:** 2017-09-07

**Authors:** Fabianne Carlesse, Anna-Paula C. Amaral, Sarah S. Gonçalves, Hemilio Xafranski, Maria-Lucia M. Lee, Victor Zecchin, Antonio S. Petrilli, Abdullah M. Al-Hatmi, Ferry Hagen, Jacques F. Meis, Arnaldo L. Colombo

**Affiliations:** 1 Oncology Pediatric Institute (IOP-GRAACC), Escola Paulista de Medicina, Federal University of Sao Paulo (UNIFESP), Sao Paulo, Brazil; 2 Department of Pediatrics, Escola Paulista de Medicina, Federal University of Sao Paulo (UNIFESP), Sao Paulo, Brazil; 3 Division of Infectious Diseases, Escola Paulista de Medicina, Federal University of Sao Paulo (UNIFESP), Rua Pedro de Toledo, 669 e 5° -andar, Sao Paulo, CEP 04039-032 Brazil; 4 Westerdijk Fungal Biodiversity Centre, Utrecht, The Netherlands; 5 Center of Expertise in Mycology Radboudumc/CWZ, Nijmegen, The Netherlands; 6 Department of Medical Microbiology and Infectious Diseases, Canisius Wilhelmina Hospital (CWZ), Nijmegen, The Netherlands

**Keywords:** Fusariosis, *Fusarium* sp., *Fusarium oxysporum*, Catheter-related *Fusarium* fungemia, Pediatric invasive fungal infections

## Abstract

**Background:**

*Fusarium* species are widely spread in nature as plant pathogens but are also able to cause opportunistic fungal infections in humans. We report a cluster of *Fusarium oxysporum* bloodstream infections in a single pediatric cancer center.

**Methods:**

All clinical and epidemiological data related to an outbreak involving seven cases of fungemia by *Fusarium oxysporum* during October 2013 and February 2014 were analysed. All cultured isolates (*n* = 14) were identified to species level by sequencing of the *TEF1* and *RPB2* genes. Genotyping of the outbreak isolates was performed by amplified fragment length polymorphism fingerprinting.

**Results:**

In a 5-month period 7 febrile pediatric cancer patients were diagnosed with catheter-related *Fusarium oxysporum* bloodstream infections. In a time span of 11 years, only 6 other infections due to *Fusarium* were documented and all were caused by a different species, *Fusarium solani*. None of the pediatric cancer patients had neutropenia at the time of diagnosis and all became febrile within two days after catheter manipulation in a specially designed room. Extensive environmental sampling in this room and the hospital did not gave a clue to the source. The outbreak was terminated after implementation of a multidisciplinary central line insertion care bundle. All *Fusarium* strains from blood and catheter tips were genetically related by amplified fragment length polymorphism fingerprinting. All patients survived the infection after prompt catheter removal and antifungal therapy.

**Conclusion:**

A cluster with, genotypical identical, *Fusarium oxysporum* strains infecting 7 children with cancer, was most probably catheter-related. The environmental source was not discovered but strict infection control measures and catheter care terminated the outbreak.

**Electronic supplementary material:**

The online version of this article (10.1186/s13756-017-0247-3) contains supplementary material, which is available to authorized users.

## Background

Due to prolonged survival of patients with cancer, invasive fungal diseases (IFD) emerged as important cause of morbidity and mortality. In pediatric cancer patients, *Candida* species remain as the most important cause of IFD followed by invasive infections by *Aspergillus* spp. and other moulds [1–3].

Epidemiology of fungal infections varies markedly according to the geographic region. In Brazil, *Fusarium* spp. infections have emerged as major pathogens of systemic infections among cancer patients [4]. *Fusarium* species are widely spread in nature as plant pathogens and are also able to cause superficial, locally invasive and disseminated infections in humans [5]. Infection occurs mainly through airborne transmission, skin breakdown due to trauma, burns or insertion of vascular catheters [6, 7]. The clinical presentation relies mostly on the hosts’ immune status, with disseminated infections been reported especially in severe immunocompromised individuals leading to high mortality rates [6, 8].

The lack of data concerning the epidemiology and clinical aspects of invasive infections by *Fusarium* spp. in pediatric patients is notable. Here we report a cluster of *Fusarium oxysporum* bloodstream infections from seven pediatric cancer patients documented in a single Brazilian pediatric cancer center.

## Methods

We retrospectively studied all clinical and epidemiological data related to an outbreak enrolling seven cases of fungemia by *Fusarium oxysporum* occurring between October 2013 and February 2014 in the Pediatric Oncology Institute (IOP), located in Sao Paulo, Brazil. IOP is a tertiary care hospital specialized in pediatric oncology which admits 300 new cases per year. The outbreak was documented in patients who had been treated with chemotherapy at the same room used for cancer ambulatory chemotherapy. All long-term intravascular catheters are implanted in the surgery room and cared for by a group of trained nurses.

Clinical and epidemiological data were collected by a single investigator who reviewed all electronic medical records in order to complete a clinical form including information related to gender, age, underlying diseases, presence of neutropenia, exposition to invasive medical procedures and immunosuppressive drugs, imaging, culture results, treatment and clinical outcome.

During the outbreak, all catheters were removed and cultured in accordance with procedures standardized in our division based on cultures of catheter tips and material inside the reservoir. A cut-off of ≥10^3^ CFU/plate was used to differentiate significant from insignificant colonization as suggested by Brun–Buisson et al. [9, 10]. All patients were screened for putative deep-seated infection by sinus and chest CT as well as echocardiography and eye fundoscopy. After the diagnosis of fusariosis, at least two serum samples were collected from each patient to check for galactomannan levels and patients were followed for another 90 days for clinical signs and symptoms.

### Laboratory procedures

#### Isolates

All *Fusarium* spp. cultured from blood and catheter samples (swab, flush and tip) were initially identified by morphological characteristics [11] and further identified to species level by sequencing *TEF1* and *RPB2* genes [12].

Besides clinical strains, we cultured environmental samples including air-filters, tap water samples, swab of room surfaces, and different intravenously administered drugs [13, 14]. We finally also evaluated all health care workers for the presence of onychomycosis or other superficial fungal infections.

### Molecular identification of *Fusarium* isolates

Genomic DNA of all *Fusarium* isolates was extracted with the PrepMan Ultra Sample Preparation Reagent kit (Applied Biosystems, Palo Alto, CA, USA) according to the manufacturer’s instructions. PCRs were performed for the amplification of the largest subunit of RNA polymerase (*RPB2*) and the translation elongation factor-1α (*TEF1α*) following the methods published by Salah et al. [15]. The primer pairs for *TEF1α* were EF1 & EF2 [16] and for *RPB2* were RPB2-7cR & RPB2 -5F [17]. PCR products were sequenced with the same primers used for amplification. The ABI Prism® Big DyeTM Terminator v. 3.0 Ready Reaction Cycle Sequencing Kit (Applied Biosystems) was used for sequencing PCR according to the manufacturer’s instructions. The samples were run on an ABI 3730XL automatic sequencer (Applied Biosystems). A BLAST search of *TEF1* and *RPB2* sequences against the database FUSARIUM-ID (http://isolate.fusariumdb.orgl/), the *Fusarium* database (http://www.cbs.knaw.nl/*fusarium*) and GenBank databases (www.ncbi.nlm.nih.gov) were used as an initial step to identify isolates to species and/or species complex. The *TEF1* and *RPB2* nucleotide sequences of all isolates were deposited in GenBank.

### Amplified fragment length polymorphism

Amplified fragment length polymorphism (AFLP) fingerprinting was performed as described recently [18]. Briefly, ~50 ng of genomic DNA was digested by using the HpyCH4IV and MseI restriction enzymes (New England Biolabs, Ipswich, MA, U.S.A.) and in the same reaction specific adaptors were ligated to each of the two sticky ends of the DNA fragments. The samples were after a 1 h incubation at room temperature diluted with 10 mM Tris-HCl, 1.0 μl of this diluted product was used as input for the PCR that contains the selective primers HpyCH4IV-C (5′-Flu-GTAGACTGCGTACCCGTAC-3′) and MseI-TGAG (5′-GATGAGTCCTGACTAATGAG). After the PCR was performed, the amplicons were 20× diluted using ddH_2_O and 1.0 μl of it was mixed with 8.9 μl ddH_2_O and 0.1 μl LIZ600 internal size marker (Promega, Leiden, The Netherlands) followed by a heating step for 1 min at 100 °C. Fragment analysis was performed on an ABI3500xL Genetic Analyzer (Applied Biosystems, Foster City, CA, U.S.A.) according to the manufacturer’s instructions. Subsequently, the raw data was imported into Bionumerics v7.5 (Applied Maths, Sint Martems-Latum, Belgium), after processing the data a dendrogram was generated by using the UPGMA algorithm.

## Results

From October 2013 to February 2014 we were caught by surprise with the occurrence of seven episodes of microbiologic documented catheter-related *F. oxysporum* bloodstream infections (CR-BSI) in our division. This finding led us to believe that we were facing an outbreak because only six cases of invasive fusariosis had been diagnosed in our hospital in the 11 years previously, all caused by *Fusarium solani* species complex strains.

Demographic and clinical data of all seven patients are depictured in Table 1. Ages ranged from 0 to 8 years (3 males), 6 out 7 were treated with chemotherapy for solid tumors, and one for acute lymphoid leukemia (ALL). Two patients (solid tumors) had underwent an autologous stem cell transplantation more than 14 days before and none of the patients received corticosteroids or other immunosuppressive agents. Only one patient had a neutrophil count below 500 cells/mm^3^ at the moment of the infection. Fever was the only trigger to collect blood cultures and vascular catheters were removed promptly at the time fungemia was identified. The six intravascular devices removed included 5 totally surgically implanted (Porth-a-Cath) catheters and one peripherally inserted catheter (PICC). All fungemic episodes were documented following 2 days after the last catheter puncture by the nurse. No single patient had any additional skin breakdown or onychomycosis that could explain the portal of entrance for *Fusarium* spp. All catheter tips, reservoir and blood cultures collected through the CVC of the 7 patients yielded *Fusarium oxysporum*. Although they had no signs or symptoms of any organ involvement, further laboratorial investigation was conducted to exclude fungal invasion of target organs. All children had a chest computer tomography (CT), sinus magnetic resonance image, eye fundoscopy and echocardiography done without any evidence of a deep-seated fungal infection. All serum samples were galactomannan negative. Catheters were promptly removed after blood cultures became positive and all patients had good clinical response to antifungal therapy. Oral voriconazole was the therapy of choice for six patients (7 mg/kg/dose) and adequate serum levels (≥1 mg/L) were measured in all patients. One child was treated with liposomal amphotericin B (5 mg/kg/day) due to age restrictions (9 months) for using voriconazole.

**Table 1:** Demographics and clinical characteristics of 7 patients with catheter-related fungemia due to *Fusarium oxysporum*

Case	Underlying diseases	HSCT	Age (years)	Gender	Neutropenia	CVC	GMI	Time interval between CVC placement and infection	Chest CT	ECHO	Fundoscopic exam	Therapy	Duration (days)	Outcome
1	Solid tumor	Auto	3	Male	Yes	Port-a-cath	Negative	665 days	Normal	Normal	Normal	Voriconazole	21	Alive
2	Solid tumor	No	2	Female	No	Port a cath	Negative	89 days	Normal	Normal	Normal	Voriconazole	21	Alive
3	Solid tumor	No	1	Male	No	Port a cath	Negative	114 days	Normal	Normal	Normal	Voriconazole	21	Alive
4	Solid tumor	No	9 months	Female	No	Port a cath	Negative	125 days	Normal	Normal	Normal	L-Ampho B	16	Alive
5	Solid tumor	Auto	1	Female	No	Port a cath	Negative	112 days	Normal	Normal	Normal	Voriconazole	21	Alive
6	Solid tumor	No	2	Female	No	Port a cath	Negative	282 days	Normal	Normal	Normal	Voriconazole	21	Alive
7	ALL in remission	No	8	Male	No	PICC	Negative	158 days	Normal	Normal	Normal	Voriconazole	14	Alive

*ALL* acute lymphoblastic leukemia, *Auto* Autologous, *CVC* central venous catheter, *PICC* peripherally inserted central catheter, *GMI* galactomannan index, *ECHO* echocardiography, *Chest CT* chest computed tomography, *L-Ampho B* liposomal amphotericin B

It is important to note that during the whole period this cluster occurred, the hospital building was being enlarged, counting with an extensive construction area.

All environmental samples and different intravenous preparations in search for sources of *Fusarium* spp. were negative. Healthcare workers who had previous contact with the patients were checked for the presence of any cutaneous or nail lesions suggestive of fungal infections but nothing was found.

### Molecular identification of *Fusarium* isolates

A total of 16 *Fusarium* spp. clinical isolates were identified to genus level by colony and microscopic characteristics of the isolates. Upon molecular identification, based on *TEF1* and *RPB2* partial genes analysis, all *Fusarium* isolates were found to be members of the *F. oxysporum* species complex. All environmental samples were culture negative.

A phylogenetic tree (Additional file 1: Figure S1) was constructed with a total of 20 sequences for two genes, with three strains from the *Fusarium solani* species complex (FSSC) i.e. *F. falciforme* (NRRL32542), *F. keratoplasticum* (FRC-S2477) and *F. petroliphilum* (NRRL32856) as outgroup. The generated tree was separated into two clades. Clade 1 included all members of the *F. oxysporum* species complex (FOSC) and clade 2 represented the *F. solani* species complex (FSSC), the final result of the identification process was that 16 isolates were confirmed as members of FOSC.

### Typing results by amplified fragment length polymorphism

**Fig. 1:**
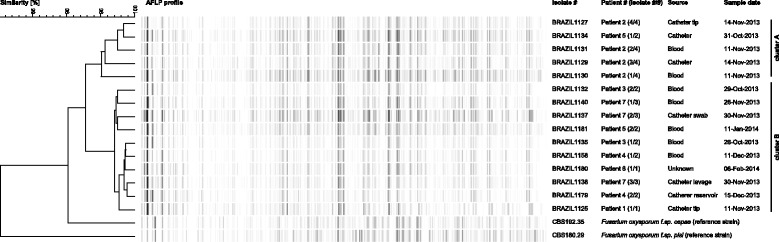
AFLP fingerprint analysis was performed on 16 clinical *Fusarium oxysporum* species complex isolates. The dendrogram was generated by using the Pearson correlation similarity coefficient followed by single linkage cluster analysis

All *Fusarium* strains obtained from blood and catheter tips were considered genetically related by AFLP fingerprinting, suggesting that all isolates had a common source. Cluster analysis revealed the presence of two clusters, one with a 95% similarity that includes isolates BRAZIL 1127 (Patient 2 catheter tip culture), 1129 (Patient 2 reservoir culture), 1131 (Patient 2 blood culture) and 1134 (Patient 3 catheter tip culture), and a second one with all other Brazilian isolates that exhibited similarity of 97% (Fig. 1). The reference *F. oxysporum* isolates CBS192.31 and CBS180.29, had a similarity of 90% and 80%, respectively, to the Brazilian isolates.

## Discussion

In Brazil, invasive fusariosis is the most common cause of systemic mould infections in hematologic cancer patients [4] and has been associated with a poor prognosis [19]. There is a gap in knowledge with regard to the epidemiology of fusariosis in pediatric patients. Most papers represent single case reports, with the exception of two outbreaks that were also documented in Brazilian medical centers and a review paper [20–22]. Information provided by the largest pediatric series of fusariosis published in the medical literature shows that the most common underlying condition was leukemia. Lung, blood and skin were the most commonly reported sites of infection and mortality rates are usually higher than 50% [22].

There is a consensus in the literature that the respiratory tract is the main portal of entry for this fungal infection, followed by skin breakdown due trauma and/or burns [23–31]. Despite some reports suggesting that fusariosis may also be acquired by contamination of a central venous catheter, the real prevalence and the natural history of intravascular catheter related fusariosis remains unclear [32–36].

Velasco and colleagues reported four immunocompromised children between 4 and 12 years, having neuroblastoma (*n* = 1) or hematologic malignancies (*n* = 3) who developed catheter-related *Fusarium* spp. infections without neutropenia. Fungemia was followed by skin lesions in only one patient and there was no clinical or radiological evidence of respiratory tract involvement. All patients survived after cathether removal and treatment with conventional amphotericin B [21].

Recently, Colombo et al. [35] described a case of a 13-year-old boy in Italy diagnosed with ALL readmitted 1 month after the start of chemotherapy due to intermittent fever of unknown origin, without any clinical or radiological evidence of invasive infection. Patient had his Broviac central venous catheter removed and blood cultures taken from the CVC were positive only for *F. solani* species complex. In addition, scanning electron microscopy of the catheter tip demonstrated the formation of a large mycelium in the catheter lumen that yielded a positive culture of *F. solani* complex. This finding represents a strong support for the hypothesis that fungemia was related to fungal colonization of the catheter. Fungemia resolved without any evidence of deep-seated infection after prompt CVC removal and therapy with voriconazole.

We report an outbreak of *F. oxysporum* infection enrolling 6 children with solid cancers and one with ALL in complete remission without persistent neutropenia. Fever was the only clinical manifestation of infection and the trigger to collect blood cultures in all episodes. Patients were carefully examined, and no single event of fungal deep tissue infection was documented. Finally, in the absence of respiratory infection as well as any type of skin breakdown that could be the port of entry, we are convinced that the intravascular catheter was the source of all episodes of *F. oxysporum*. Indeed, by typing all isolates with AFLP we confirmed our hypothesis that the 7 cases of *F oxysporum* were related, and all cultures of catheter tips and reservoirs had similar or identical genotypes to isolates obtained from blood cultures. Because all environmental investigations were negative for the presence of *Fusarium* spp., we were not able to identify the presumed common source of all infections. On the other hand, we may not exclude the possibility that medical devices were colonized by *Fusarium* during inappropriate management of intravascular catheters by health care workers or by the contamination of intravascular fluids.

Disseminated fusariosis is a difficult to treat fungal infection with an overall mortality ranging between 50% and 80% mostly due to uncontrolled hematologic malignant disease, prolonged neutropenia and the exposition to corticosteroids [19, 37]. Indeed, during a recent outbreak among children with cancer, fungemia and deep-seated infections due to *Fusarium* the mortality rate was 50% [20].

In contrast, in the present series, and as documented by previous reports of catheter related fusariosis [21, 33], we observed a complete clinical recovery of all patients after removal of the intravascular catheter and initiation of appropriate antifungal therapy. The good outcome documented with our patients may be explained also because there was no neutropenia or exposition to corticosteroids at the time they developed fusariosis.

*F. oxysporum* was the etiologic agent responsible for all fungemias documented in the present outbreak. This finding is in accordance with previous reports where seven out ten cases of *Fusarium* spp. fungemias and intravascular catheter related infections were due non-*solani Fusarium* species [21, 23, 33–36, 38].

Measures taken to control the outbreak were lowering the level of humidity in the CVC storage room and the implementation of a multidisciplinary quality-improvement intervention, including a central line insertion care bundle, consisting of hand hygiene, maximal sterile barrier use upon insertion and use of chlorhexidine skin disinfection. After the introduction of these actions, no further cases were noted.

In conclusion, *Fusarium* fungemia may be acquired by intravascular catheter colonization without any further deep-seated infections. In case of *Fusarium* outbreaks, especially involving non-*F. solani* species, clinicians should be aware that contamination of central venous catheter may be the potential source of infection.

## Conclusions

We described 7 episodes of *Fusarium oxysporum* fungemia where we presented strong clinical and laboratorial documentation supporting the hypothesis that the portal of entry for all cases of fusariosis was the intravascular central catheter. In case of *Fusarium* outbreaks, especially involving non-*F. solani* species, clinicians should be aware that contamination of central venous catheter may be a potential source. Finally, we added information suggesting that the clinical outcome of catheter related fungemia due to *Fusarium* may be favorable in patients without neutropenia who were submitted to prompt CVC removal and early initiation of antifungal therapy.

## Additional file

Additional file 1: Figure S1. Phylogenetic tree resulting from RAxML analysis for the RPB2 and TEF1genes (values of 85% for maximum likelihood are shown). List of the 16 isolates examined in this study (Brazil) and controls. (TIFF 330 kb)

## Abbreviations

AFLP: Amplified fragment length polymorphism
ALL: Acute lymphoid leukemia
CFU: Colony forming unit
CR-BSI: Catheter-related blood stream infections
CT: Computer tomography
CVC: Central venous catheter
FOSC: Fusarium solani species complex
FSSC: Fusarium solani species complex
IFD: Invasive fungal diseases
PICC: Peripherally inserted central catheter

## References

[1] Castagnola E, Caviglia I, Pistorio A, Fioredda F, Micalizzi C, Viscoli C (2005). Bloodstream infections and invasive mycoses in children undergoing acute leukaemia treatment: a 13-year experience at a single Italian institution. Eur J Cancer.

[2] Castagnola E, Cesaro S, Giacchino M, Livadiotti S, Tucci F, Zanazzo G (2006). Fungal infections in children with cancer: a prospective, multicenter surveillance study. Pediatr Infect Dis J.

[3] Hale KA, Shaw PJ, Dalla-Pozza L, MacIntyre CR, Isaacs D, Sorrell TC (2010). Epidemiology of paediatric invasive fungal infections and a case-control study of risk factors in acute leukaemia or post stem cell transplant. Br J Haematol.

[4] Nucci M, Garnica M, Gloria AB, Lehugeur DS, Dias VC, Palma LC (2013). Invasive fungal diseases in haematopoietic cell transplant recipients and in patients with acute myeloid leukaemia or myelodysplasia in Brazil. Clin Microbiol Infect.

[5] Al-Hatmi AM, Meis JF, de Hoog GS (2016). *Fusarium*: Molecular diversity and intrinsic drug resistance. PLoS Pathog.

[6] Nucci M, Anaissie EJ, Queiroz-Telles F, Martins CA, Trabasso P, Solza C (2003). Outcome predictors of 84 patients with hematologic malignancies and *Fusarium* infection. Cancer.

[7] Raad I, Tarrand J, Hanna H, Albitar M, Janssen E, Boktour M (2002). Epidemiology, molecular mycology, and environmental sources of *Fusarium* infection in patients with cancer. Infect Control Hosp Epidemiol.

[8] Dignani MC, Anaissie E (2004). Human fusariosis. Clin Microbiol Infect.

[9] Douard MC, Arlet G, Longuet P, Troje C, Rouveau M, Ponscarme D (1999). Diagnosis of venous access port-related infections. Clin Infect Dis.

[10] Brun-Buisson C, Abrouk F, Legrand P, Huet Y, Larabi S, Rapin M (1987). Diagnosis of central venous catheter-related sepsis. Critical level of quantitative tip cultures. Arch Intern Med.

[11] Azor M, Gene J, Cano J, Manikandan P, Venkatapathy N, Guarro J (2009). Less-frequent *Fusarium* species of clinical interest: correlation between morphological and molecular identification and antifungal susceptibility. J Clin Microbiol.

[12] Al-Hatmi AM, Hagen F, Menken SB, Meis JF, de Hoog GS (2016). Global molecular epidemiology and genetic diversity of *Fusarium*, a significant emerging group of human opportunists from 1958 to 2015. Emerg Microbes Infect.

[13] Mesquita-Rocha S, Godoy-Martinez PC, Goncalves SS, Urrutia MD, Carlesse F, Seber A (2013). The water supply system as a potential source of fungal infection in paediatric haematopoietic stem cell units. BMC Infect Dis.

[14] Edel-Hermann V, Sautour M, Gautheron N, Laurent J, Aho S, Bonnin A (2016). A clonal lineage of *Fusarium oxysporum* circulates in the tap water of different French hospitals. Appl Environ Microbiol.

[15] Salah H, Al-Hatmi AM, Theelen B, Abukamar M, Hashim S, van Diepeningen AD (2015). Phylogenetic diversity of human pathogenic *Fusarium* and emergence of uncommon virulent species. J Inf Secur.

[16] O'Donnell K, Kistler HC, Cigelnik E, Ploetz RC (1998). Multiple evolutionary origins of the fungus causing Panama disease of banana: concordant evidence from nuclear and mitochondrial gene genealogies. Proc Natl Acad Sci U S A.

[17] Reeb V, Lutzoni F, Roux C (2004). Contribution of RPB2 to multilocus phylogenetic studies of the euascomycetes (Pezizomycotina, Fungi) with special emphasis on the lichen-forming Acarosporaceae and evolution of polyspory. Mol Phylogenet Evol.

[18] Al-Hatmi AM, Mirabolfathy M, Hagen F, Normand AC, Stielow JB, Karami-Osbo R (2016). DNA barcoding, MALDI-TOF, and AFLP data support *Fusarium ficicrescens* as a distinct species within the *Fusarium fujikuroi* species complex. Fungal Biol.

[19] Nucci M, Anaissie E (2007). *Fusarium* infections in immunocompromised patients. Clin Microbiol Rev.

[20] Litvinov N, da Silva MT, van der Heijden IM, Graca MG, Marques de OL, Fu L (2015). An outbreak of invasive fusariosis in a children’s cancer hospital. Clin Microbiol Infect.

[21] Velasco E, Martins CA, Nucci M (1995). Successful treatment of catheter-related fusarial infection in immunocompromised children. Eur J Clin Microbiol Infect Dis.

[22] Schwartz KL, Sheffield H, Richardson SE, Sung L, Morris SK (2015). Invasive fusariosis: A single pediatric center 15-year experience. J Pediatric Infect Dis Soc.

[23] Chaulk CP, Smith RW, Feagler JR, Verdirame J, Commers JR (1986). Fungemia due to *Fusarium solani* in an immunocompromised child. Pediatr Infect Dis.

[24] Schneller FR, Gulati SC, Cunningham IB, O'Reilly RJ, Schmitt HJ, Clarkson BD (1990). *Fusarium* infections in patients with hematologic malignancies. Leuk Res.

[25] Okuda C, Ito M, Sato Y, Oka K, Hotchi M (1987). Disseminated cutaneous *Fusarium* infection with vascular invasion in a leukemic patient. J Med Vet Mycol.

[26] Lozano M, Ribera JM, Puig J, Rives A, Sierra J, Granena A (1990). *Fusarium solani* bronchopneumonia in a patient with acute myeloblastic leukemia. Enferm Infecc Microbiol Clin.

[27] Alvarez-Franco M, Reyes-Mugica M, Paller AS (1992). Cutaneous *Fusarium* infection in an adolescent with acute leukemia. Pediatr Dermatol.

[28] Nucci M, Anaissie E (2002). Cutaneous infection by *Fusarium* species in healthy and immunocompromised hosts: implications for diagnosis and management. Clin Infect Dis.

[29] Nucci M, Varon AG, Garnica M, Akiti T, Barreiros G, Trope BM (2013). Increased incidence of invasive fusariosis with cutaneous portal of entry, Brazil. Emerg Infect Dis.

[30] Varon AG, Nouer SA, Barreiros G, Trope BM, Magalhaes F, Akiti T (2014). Superficial skin lesions positive for *Fusarium* are associated with subsequent development of invasive fusariosis. J Inf Secur.

[31] Boutati EI, Anaissie EJ (1997). *Fusarium*, a significant emerging pathogen in patients with hematologic malignancy: ten years’ experience at a cancer center and implications for management. Blood.

[32] Nucci M, Marr KA, Vehreschild MJ, de Souza CA, Velasco E, Cappellano P (2014). Improvement in the outcome of invasive fusariosis in the last decade. Clin Microbiol Infect.

[33] Raad I, Hachem R (1995). Treatment of central venous catheter-related fungemia due to Fusarium oxysporum. Clin Infect Dis.

[34] Kiehn TE, Nelson PE, Bernard EM, Edwards FF, Koziner B, Armstrong D (1985). Catheter-associated fungemia caused by *Fusarium chlamydosporum* in a patient with lymphocytic lymphoma. J Clin Microbiol.

[35] Colombo A, Maccari G, Congiu T, Basso P, Baj A, Toniolo A (2013). Colonization of a central venous catheter by the hyaline fungus F*usarium solani* species complex: A case report and SEM imaging. Case Rep Med.

[36] Eljaschewitsch J, Sandfort J, Tintelnot K, Horbach I, Ruf B (1996). Port-a-cath-related *Fusarium oxysporum* infection in an HIV-infected patient: treatment with liposomal amphotericin B. Mycoses.

[37] Tortorano AM, Richardson M, Roilides E, Diepeningen AV, Caira M, Munoz P (2014). ESCMID and ECMM joint guidelines on diagnosis and management of hyalohyphomycosis: *Fusarium* spp., *Scedosporium* spp. and others. Clin Microbiol Infect.

[38] Ammari LK, Puck JM, McGowan KL (1993). Catheter-related *Fusarium solani* fungemia and pulmonary infection in a patient with leukemia in remission. Clin Infect Dis.

