# Supplementary figures and images for: Outbreak of Fusarium oxysporum infections in children with cancer: an experience with 7 episodes of catheter-related fungemia
Source: Antimicrob Resist Infect Control. 2017 Sep 7;6:93. doi: 10.1186/s13756-017-0247-3 (PMC5588724; doi:10.1186/s13756-017-0247-3)

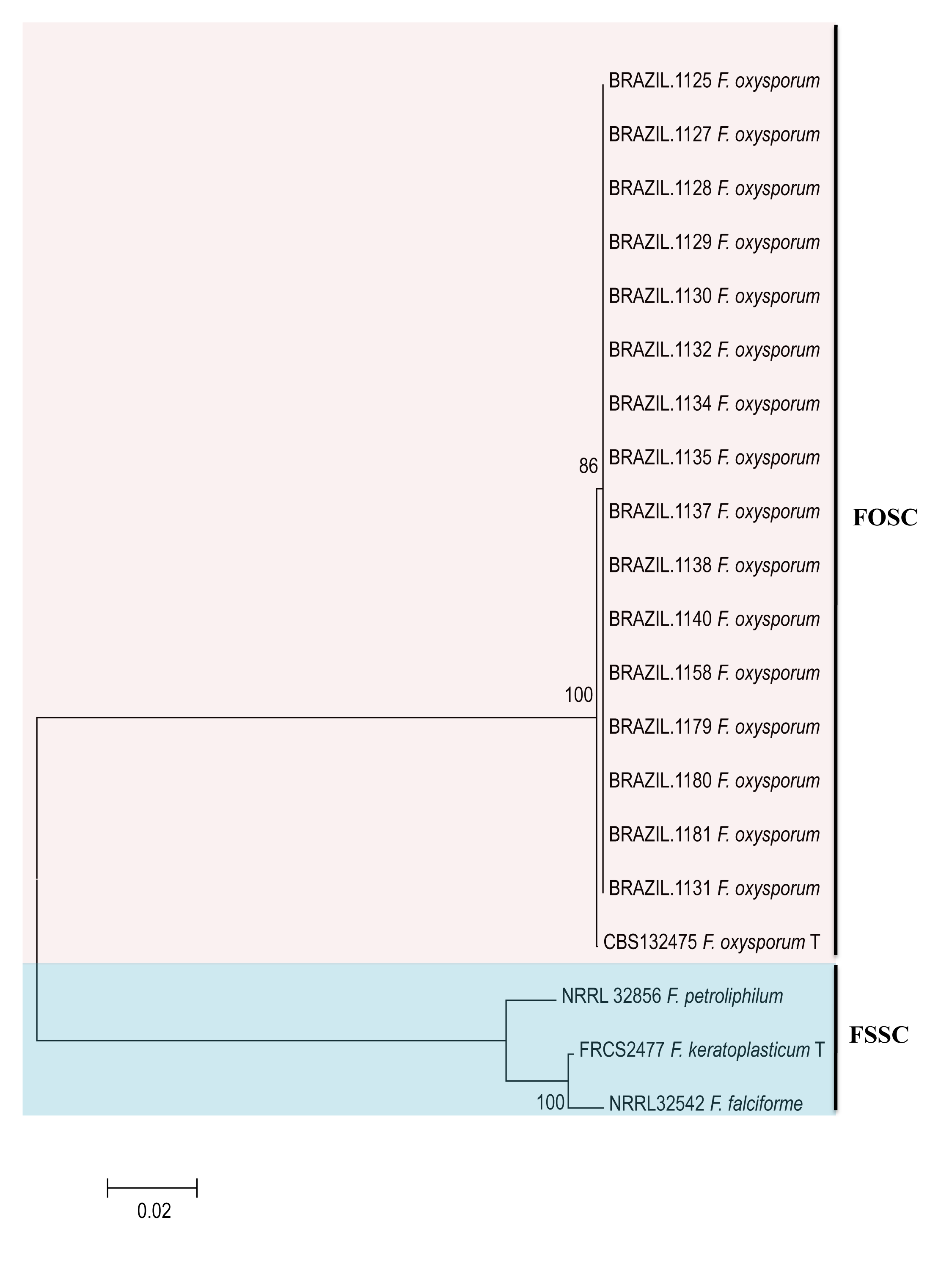

Supplement: Additional file 1: Figure S1. — Phylogenetic tree resulting from RAxML analysis for the RPB2 and TEF1genes (values of 85% for maximum likelihood are shown). List of the 16 isolates examined in this study (Brazil) and controls. (TIFF 330 kb) [file 13756_2017_247_MOESM1_ESM.tif]
